# Splanchnic nerve block via transdiscal and paraspinal approach in the treatment of pain in advanced pancreatic cancer: A randomized controlled trial

**DOI:** 10.1097/MD.0000000000044250

**Published:** 2025-09-19

**Authors:** Lingyun Luo, Xintian Cao, Mizhen Qiu, Mengye Zhu, Yi Yan, Daying Zhang, Xuexue Zhang

**Affiliations:** a Department of Anesthesiology, Jiangxi Cancer Hospital, The Second Affiliated Hospital of Nanchang Medical College, Nanchang, China; b Department of Pain Medicine, The First Affiliated Hospital of Nanchang University, Nanchang, China; c JXHC Key Laboratory of Neuropathic Pain (The First Affiliated Hospital of Nanchang University), Nanchang, China.

**Keywords:** cancerous pain, pancreatic cancer, paraspinal, splanchnic nerve block, transdiscal

## Abstract

**Background:**

This randomized controlled clinical trial aims to assess the clinical effect of splanchnic nerve block through transdiscal and paraspinal approaches in treating pain from advanced pancreatic cancer.

**Methods:**

Patients with progressive pancreatic cancer pain who were treated in the Pain Department of the First Affiliated Hospital of Nanchang University from December 2021 to March 2023 were selected. According to the inclusion and exclusion criteria, 34 patients were randomly divided into a transdiscal group (n = 17) and a paraspinal group (n = 17). The patient’s intraoperative puncture time and the number of digital subtraction angiography fluoroscopy exposures were recorded. The Visual Analogue Scale (VAS), patient satisfaction scale, Karnofsky score, morphine consumption, and occurrence of adverse events were recorded preoperatively and 1 day, 3 days, 1 week, 1 month, and 3 months after surgery.

**Results:**

A total of 14 patients were followed up in the transdiscal group, and 15 patients were followed up in the paraspinal group. There was no significant difference in the general condition between the 2 groups before the operation. The puncture time and number of fluoroscopies in the transdiscal group were significantly less than in the paraspinal group (*P* < .001). There was no significant difference in VAS, patient satisfaction scale, or Karnofsky score between the 2 groups at each postoperative time point. In the same group, the VAS scores at each time point after operation were significantly lower than those before operation (*P* < .001). The 2 groups had no significant difference in the total incidence of complications.

**Conclusion:**

Splanchnic nerve block paraspinal had a significant effect on the treatment of advanced pain in pancreatic cancer patients. However, transdiscal is equally effective, the positioning is more accurate, the operation is simpler, and it is a better choice.

## 1. Introduction

Pancreatic cancer is one of the most common malignant tumors,^[1]^ and its morbidity and mortality are rising. In 2015, there were 95,000 new cases of pancreatic cancer in China, ranking 10th in the incidence of cancer in China. In addition, it is highly malignant, but its 5-year relative survival rate is only 7.2%, which is the lowest among all cancers.^[2]^ Both local invasion and distant metastasis can occur in the early stages of cancer development.^[3]^ The pancreas is a retroperitoneal organ containing an extensive nerve plexus rich in neural tissue. Tumor cells easily infiltrate peripheral nerves, causing intractable pain. Previous data showed that about 90% of clinically diagnosed pancreatic cancers are in advanced stages, with large tumors or metastases. In addition, 75% of patients with advanced cancer experience cancer pain. This pathological pain reduces patients’ quality of life and induces negative emotions such as anxiety and depression. Therefore, besides tumor-related radical treatment or palliative treatments,^[4]^ active and adequate analgesia is of great significance.^[5]^ Celiac plexus block (CPB) effectively treats advanced cancer pain in upper abdominal malignant tumors.^[6]^ Kappis first proposed percutaneous celiac plexus destruction in 1919.^[7]^ Since then, scholars worldwide have studied the puncture approach of CPB, improving the curative effect and reducing complications.^[8]^ Although there is still controversy among scholars worldwide on improving patients’ quality of life and prolonging survival time, there is a consensus that early intervention can significantly relieve pain in advanced upper abdominal malignant tumors and significantly reduce the dosage of morphine drugs.^[9,10]^ However, tumor infiltration, operation, and ascites can change the anatomical position of the celiac plexus, affect drug diffusion, and increase the risk of puncture.

The splanchnic nerve is the primary origin of the celiac plexus, which consists of the nociceptive afferent fibers of the upper abdominal parenchymatous organ and is situated posterior to the diaphragmatic crura.^[11]^ The posterior space of the diaphragmatic crura is a potential triangular space where the visceral nerves can be in complete contact with the alcohol, and the damage effect is almost unaffected by the surrounding structure.^[12,13]^ In recent years, some experiments by Shwita^[14]^ have shown that the splanchnic nerve block (SNB) has a better analgesic effect and fewer complications than CPB.^[15]^ The primary puncture method for SNB is the transdiscal or paraspinal approach.^[16]^ The traditional SNB is a paraspinal approach prone to organ and pleural injury. In 2003, Plancarte-Sanchez^[17]^ first proposed the transdiscal approach, which can reduce the incidence of pneumothorax and other complications.

With different surgical methods, there are some differences in the curative effect. The advantages and disadvantages of these 2 surgical methods are not clear. Thus, we compare the clinical efficacy of SNB via transdiscal and paraspinal approach in the treatment of advanced pancreatic cancer pain, and the intraoperative puncture time and exposure times of digital subtraction radiography system Digital subtraction angiography (DSA) were recorded to provide reference for clinical application.

## 2. Materials and methods

### 2.1. Study design and patients

This randomized study was approved by the Ethics Committee of the First Affiliated Hospital of Nanchang University (Protocol No: IIT2021-12-023). Advanced pancreatic cancer patients treated in our hospital with intractable pain from December 2021 to March 2023 were included. Inclusion criteria included: diagnosis of abdominal pain and referred pain of the back that was secondary to cancer, visual analogue scale (VAS) is still >5 after analgesic treatment with opioids and auxiliary drugs, Pathological diagnosis of pancreatic cancer (stage III or IV). The main exclusion criteria were as follows: abdominal or back pain not related to cancer in the upper abdomen (e.g., metastases of bone or other organs, especially pelvic organs), loss to follow-up after the procedure, patients with severe abnormal Respiratory and circulatory system function, puncture site infection, and those with structural abnormalities of the spine or transdiscal disc. Besides, patients with coagulation dysfunction, those with alcohol and iodine allergies, inability to cooperate with treatment were also excluded. The operation method, expected effect, and possible complications and adverse reactions were explained to the patients and their families before the operation. Inform the patient how to prepare for the assessment of VAS, patient satisfaction scale (PSS), and karnofsky score (KS),^[18]^ so we can follow-up by phone. Afterward, the patients or their families signed the informed consent forms.

Sample size calculation was completed using SAS version 9.0. Using 2 independent proportions test (inequality) with a power of 90% at a 5% significance level, 28 patients were required. Allowing for potential dropout, a total of 34 patients were included.^[19]^ Two independent sample Student *t*-test was used to detect the minimum effect size Cohen D = 0.7. Patients were assigned to the transdiscal and paraspinal approaches for surgical treatment using a random number table method. Independent researchers were responsible for the collection of experimental data, and the data management committee was responsible for the management.

### 2.2. Methods

Preoperative preparation: 2000 mL of daily rehydration for 3 days before surgery. CT scan was also performed before surgery, and the needle depth and puncture angle were calculated when the needle tip reached the best position. Routine examination and iodine allergy tests were performed to rule out surgical contraindications. In addition, the patient underwent fasting for 2 hours before surgery and did not have analgesics on the day of operation. Continue to maintain the previous dose after surgery.

**Figure 1.**
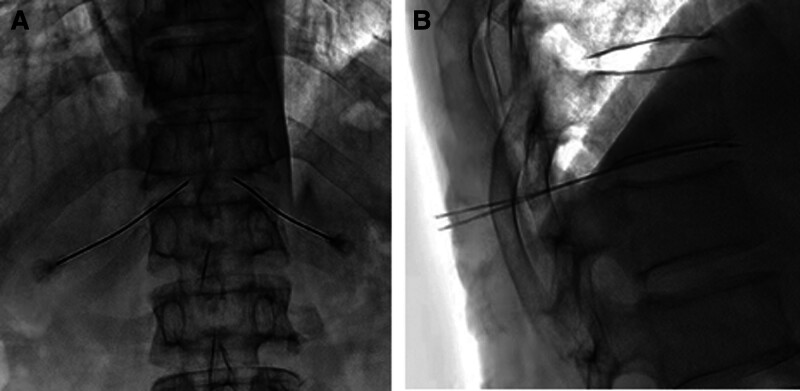
Position of the needle via transdiscal approach. (A) Anteroposterior view: the needle tip was located in the T11 to 12 transdiscal. (B) Lateral view: the needle tip was at the anterior edge of the transdiscal of T11 to 12.

The venous passage was opened, and there was continuous low-flow oxygen inhalation. ECG, blood pressure, heart rate, and oxygen saturation were monitored. The patient had soft pillows under the waist in the prone position. In addition, routine disinfection of the surgical field was performed. In the transdiscal group, 1% lidocaine local anesthesia was followed by DSA image-guided long 9.7cm and bare-end 5mm radiofrequency needle to identify the path according to the image (T11–12 transdiscal bilateral approach) and execute the puncture. According to the puncture path and angle designed before the operation, the puncture needle enters the boundary between the lateral side of the lamina and the superior articular process, we use the anterolateral X-ray to determine the position of the needle tip, and the puncture path does not pass through the spinal canal and laterally through the transdiscal to the best position as determined before the operation: laterally anterior to the ipsilateral abdominal aorta. Resistance increases as it reaches the disc and disappears after a breakthrough. The X-ray position of the lumbar spine showed that the needle tip was located in the T11 to 12 transdiscal (Fig. 1A). On the lateral film, the tip of the needle was at the anterior edge of the transdiscal of T11-12 (Fig. 1B). DSA 3D scanning is performed to observe whether the needle position reaches the predetermined position and whether there is a puncture injury. During the puncture process, the patient was repeatedly asked if there was any discomfort in the chest, abdomen, and lower extremities to prevent the spinal cord, nerve roots, blood vessels, and other injuries and pneumothorax.

**Figure 2.**
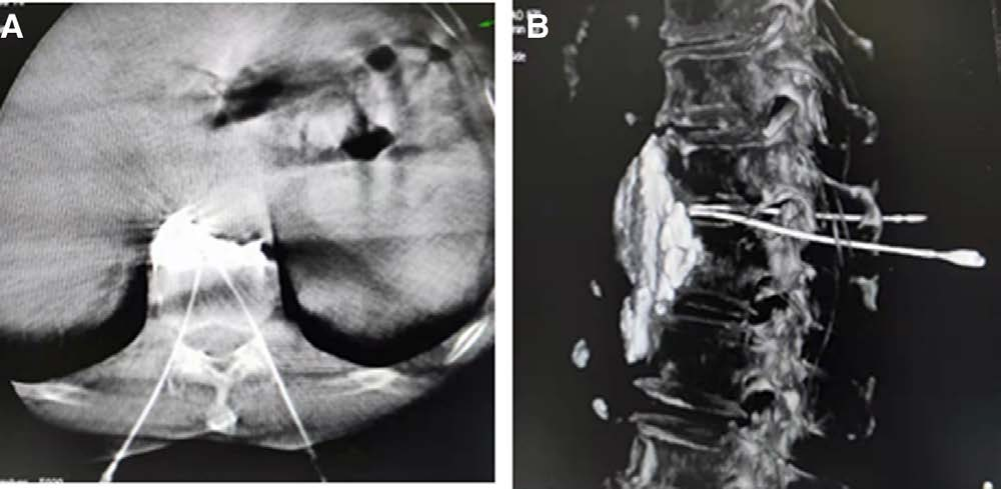
Spread of the contrast agent via transdiscal approach. (A) DSA transverse position of T11 to 12 transdiscal. (B) DSA 3D reconstruction image can show that the contrast medium was distributed in bilateral prevertebral tissue. DSA = digital subtraction angiography

Furthermore, the syringe withdrawal does not contain blood, gas, or liquid. Then, a contrast agent mixture of 5 mL iodixanol and 5 mL 2% lidocaine was extracted for injection contrast treatment. The contrast medium was distributed in bilateral prevertebral tissues (Fig. 2A and B).

**Figure 3.**
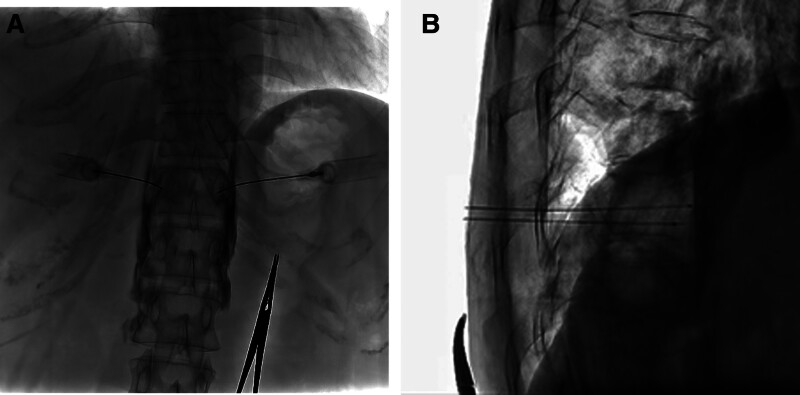
Position of the needle via paraspinal approach. (A) Anteroposterior view: the needle tip is located at the upper 1/2 of the T11 vertebral body. (B) Lateral view: the tip of the needle was at the 1/3 lateral edge of the anterior vertebral body.

**Figure 4.**
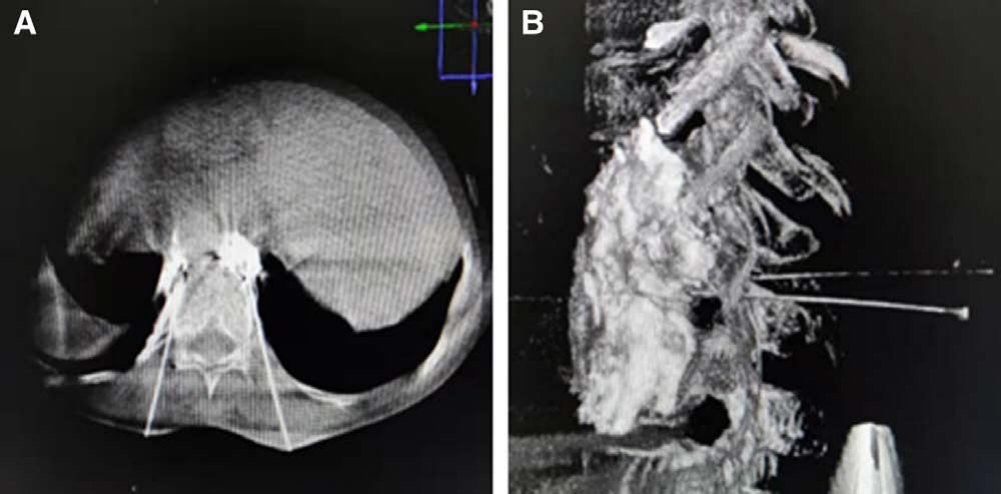
Spread of the contrast agent via paraspinal approach. (A) DSA transverse position of T11 vertebral body. (B) In the 3-dimensional reconstruction image of DSA, it can be seen that the contrast medium was distributed in the bilateral prevertebral tissue. DSA = digital subtraction angiography.

On the other hand, the paraspinal group was punctured from the upper 1/2 of the T11 vertebral body (Fig. 3A) to the 1/3 lateral edges of the anterior vertebral body according to the image mark under the same condition (Fig. 3B). DSA 3D scanning is performed to observe whether the needle position reaches the predetermined position and whether there is a puncture injury. During the puncture, the patient was repeatedly asked if discomfort was in the chest, abdomen, and lower extremities to prevent the spinal cord, nerve roots, blood vessels, other injuries, and pneumothorax. Moreover, the syringe withdrawal does not contain blood, gas, or liquid. Then, they were treated with a contrast medium mixture containing 5 mL iodixanol and 5 mL of 2% lidocaine for injection contrast treatment. The contrast medium was distributed in bilateral prevertebral tissue (Fig. 4A and B). The diagnostic block was positive when the patient’s VAS score (0–10, 0: painless, 10: pain) dropped to 50% before the operation. Then, both sides were injected with 5 mL of 99.5% absolute ethanol (total 10 mL). Before pulling the needle, 1 mL saline was injected into the puncture needle to prevent the flow of alcohol, followed by pressure hemostasis, incision suture, and external dressing. Vital signs were observed in the prone position for 30 minutes without abnormalities, and then the patients were sent back to the ward.

### 2.3. Observation index

Clinical efficacy standards: Pain was assessed by VAS (0–10), where 0 means no pain and 10 indicates the maximum level of intolerable pain. The functional status assessment of the patient was done using the KS, which was scored 0 to 100, with a higher number indicating better function. Patient satisfaction was evaluated using the PSS. The above indicators and daily morphine consumption (mg/day) were recorded preoperatively and 1 day, 3 days, 1 week, 1 month, and 3 months after surgery. Puncture time was recorded from the first DSA localization to the injection of contrast medium during the operation, and the number of fluoroscopies. Besides, complications such as back pain, diarrhea, hypotension, and pneumothorax were also recorded.^[19]^

### 2.4. Statistical analysis

All data were analyzed with SPSS version 22.0 (Chicago). The age, weight, VAS score, PSS, KS, Puncture time and the number of intraoperative fluoroscopies were by *t*-test. The sex and complications were by fisher exact test. Statistical significance was set at *P* <.05.

## 3. Results

Thirty-four patients were enrolled in this study, including 17 in the transdiscal group and 17 in the paraspinal group. Among them, 3 patients lost follow-up in the transdiscal group and 2 in the paraspinal group. Finally, 14 were followed up in the transdiscal group and 15 in the paraspinal group.

### 3.1. General information

All patients successfully underwent surgery, as well as the 3-month follow-up. There was no significant difference in sex, age, body weight, preoperative VAS score, Tumor Stage, tumor location, time since diagnosis (days), ECOG Performance Status Scale, History of anticancer treatment, and Preoperative daily consumption of morphine equivalent between the 2 groups (*P* >.05, Table 1).

**Table 1:** Comparison of general information between 2 groups.

	Transdiscal	Paraspinal	*P*-value
Age (year)	54.82 ± 9.19	55.53 ± 12.30	.85
Gender (male/female)	10/4	9/6	.70
Weight (kg)	52.24 ± 4.35	52.35 ± 8.73	.96
Preoperative VAS (score)	6.00 ± 0.61	5.59 ± 0.62	.06
Tumor stage			
III	5	4	.70
IV	9	11	
Tumor location			
pancreatic head	6	7	.99
pancreatic body/tail	8	8	
Time since diagnosis (d)	224.79 ± 51.04	216.73 ± 57.75	.69
ECOG performance status scale			
1	3	4	.90
2	7	6	
3	4	5	
4	0	0	
History of anticancer treatment			
Chemotherapy	11	12	.93
Surgery	5	6	
Radiotherapy	3	2	
Preoperative daily consumption of morphine equivalent, mg			
	183.35 ± 71.52	201.63 ± 58.94	.27

ECOG = Eastern Cooperative Oncology Group, VAS = visual analogue scale.

### 3.2. The puncture time and number of fluoroscopies were compared between the 2 groups

**Figure 5.**
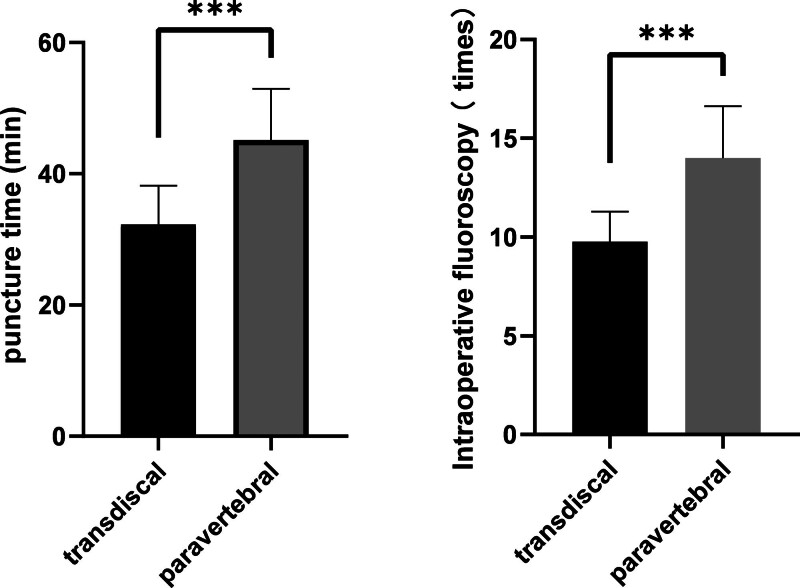
Puncture time and the number of intraoperative fluoroscopies were compared between the 2 groups. In the transdiscal approach group, the puncture time was 32.29 ± 5.92 min, and the number of intraoperative fluoroscopies was 9.77 ± 1.52. Compared with the paraspinal approach group, the puncture time (45.12 ± 7.84 min) and the number of intraoperative fluoroscopies (14.00 ± 2.62) were reduced (*** *P* <.001).

The puncture time and number of fluoroscopies in the transdiscal group were significantly lower than in the paraspinal group (*P* <.001, Fig. 5).

### 3.3. The VAS scores of the 2 groups

**Figure 6.**
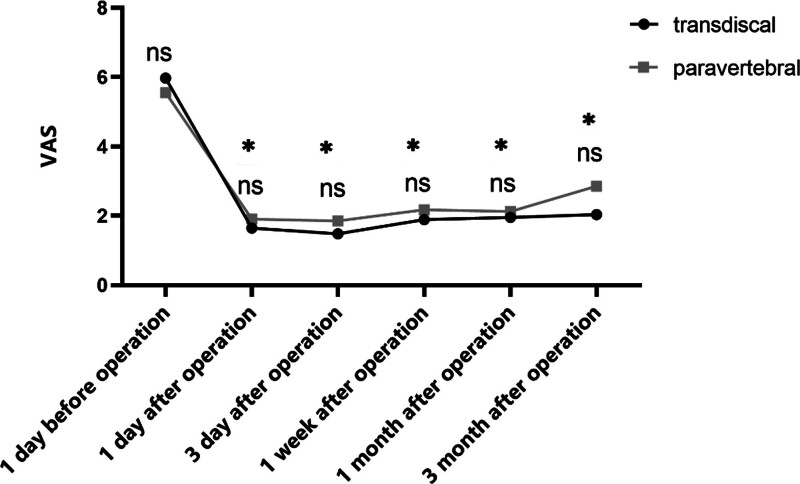
The VAS scores of the 2 groups were compared. In the transdiscal approach group, the first day after operation was 1.77 ± 0.66, the third day was 1.59 ± 0.62, the first week was 2.00 ± 0.65, 1 mo was 2.00 ± 0.43, 3 mo was 2.14 ± 0.69. In the paraspinal approach group, the first day after the operation was 2.00 ± 0.61, the third day was 2.06 ± 1.03, the first week was 2.36 ± 1.01, 1 mo was 2.27 ± 0.79, 3 mo was 3.00 ± 1.10. There was no significant difference in VAS between the 2 groups at each time point after the operation (*P* on the first day after operation was .06, the third day was .29, the first week was .19, 1 mo was .33, and 3 mo was .11). Compared with the same group before operation, there was a significant decrease in the VAS score at each time point after operation (*P* <.001). VAS = visual analogue scale.

There was no significant difference in the VAS between the 2 groups at each time point after the operation (*P* on the first day after operation was .06, the third day was .29, the first week was .19, 1 month was .33, 3 months was .11). Compared with the same group before the operation, there was a significant decrease in the VAS score at each time point after the operation (*P* <.001, Fig. 6).

### 3.4. The 2 groups’

Daily consumption of morphine equivalent, KS, and PSS were compared. There is no significant difference in the above indicators. (*P* >.05, Table 2).

**Table 2:** Comparison of VAS, daily consumption of morphine equivalent, KS, and PSS from preoperative to postoperative.

Indexes	Transdiscal	Paraspinal	*P*-value
VAS			
Baseline	6.00 ± 0.61	5.59 ± 0.62	.06
Day 1	1.77 ± 0.66	2.00 ± 0.61	.29
Day 3	1.59 ± 0.62	2.06 ± 1.03	.19
Week 1	2.00 ± 0.65	2.36 ± 1.01	.33
Month 1	2.00 ± 0.43	2.27 ± 0.79	.19
Month 3	2.14 ± 0.69	3.00 ± 1.10	.11
Daily consumption of morphine equivalent, mg			
Baseline	183.35 ± 71.52	201.63 ± 58.94	.27
Day 1	73.21 ± 63.59	75.14 ± 52.76	.68
Day 3	76.34 ± 58.73	77.23 ± 54.94	.95
Week 1	98.74 ± 47.23	93.57 ± 633.12	.67
Month 1	103.57 ± 53.48	118.95 ± 51.74	.30
Month 3	127.41 ± 63.54	139.28 ± 48.54	.47
KS			
Baseline	52.37 ± 8.65	55.45 ± 6.54	.72
Day 1	68.54 ± 7.65	66.75 ± 7.28	.63
Day 3	66.12 ± 9.28	68.36 ± 6.87	.71
Week 1	62.24 ± 6.35	65.43 ± 9.11	.59
Month 1	57.25 ± 8.57	59.43 ± 5.86	.43
Month 3	51.47 ± 7.81	53.27 ± 6.72	.64
PSS			
Baseline	2.35 ± 1.12	1.97 ± 0.76	.12
Day 1	6.82 ± 1.35	6.59 ± 1.14	.41
Day 3	6.65 ± 1.42	6.72 ± 1.23	.57
Week 1	6.75 ± 1.68	6.53 ± 1.44	.69
Month 1	6.15 ± 2.15	5.97 ± 2.36	.26
Month 3	5.76 ± 2.35	5.18 ± 2.58	.36

KS = Karnofsky score, PSS = patient satisfaction scale, VAS = visual analogue scale.

### 3.5. Complication

Our data showed that most patients had varying back or abdominal pain degrees. In the transdiscal group, the incidence of complications was 42.9% (6/14). In the paraspinal group, the incidence of complications was 33.3% (5/15). There was no significant difference in complications between the 2 groups (*P* >.05, Table 3). Besides, there were no severe complications such as spinal cord, abdominal aorta injury, pneumothorax, and drug reflux transdiscal foramen. Back pain and diarrhea patients were relieved after symptomatic treatment, and hypotension patients recovered after rapid rehydration and intravenous injection of dopamine 10 mg.

**Table 3:** Comparison of back pain, hypotension, and diarrhea.

Group	Back pain	Hypotension	Diarrhea	*P*-value
Transdiscal (n = 14)	3 (21.4%)	1 (7.1%)	2 (14.3%)	.52
Paraspinal (n = 15)	2 (13.3%)	0 (0%)	3 (20%)	

## 4. Discussion

The results showed that the puncture time of the percutaneous transdiscal approach was less than that of the paraspinal approach. This is because the anatomical path of the transdiscal approach is relatively fixed, and the puncture path does not include essential organs and pleura. The DSA was adjusted to the same plane of the anterior and posterior edge of the T11 to 12 vertebral body; the transdiscal was utterly exposed, and thus, the needle could be inserted after calculating the puncture path parallel to the transdiscal. The puncture needle enters the boundary between the lateral side of the lamina and the superior articular process. The resistance increases when the needle penetrates the transdiscal, and then the needle continues to enter the direction of the parallel transdiscal. When the needle passes through the anterior longitudinal ligament, there is a sense of breakthrough,^[20]^ which, combined with DSA guidance, can arrive quickly and accurately. As a result, time in the prone position can be reduced, and the patient tolerance can be improved^[20]^; this is consistent with Rosland report that the transdiscal approach can avoid potential pleural damage and drug reflux to transdiscal foramen or psoas major muscle, and reduce the risk of intraoperative bleeding.^[21]^

On the other hand, the paraspinal approach needle should be tried repeatedly on the surface of the vertebral body. There is no fixed anatomical path, and the angle and depth can be adjusted after multiple exposures to reach the lateral anterior edge of the vertebral body. It is difficult to confirm whether the puncture needle is between the vertebral body and the parietal pleura under fluoroscopy, and the incidence of pneumothorax is high. The puncture path is close to vascular organs such as the azygos vein and intercostal artery, which increases the risk of intraoperative bleeding injury.^[22]^ The key to the success of SNB is puncture location and the diffusion range of ethanol. The ideal analgesic effect can be achieved only by choosing the correct puncture location, a safe and reliable puncture path and the full infiltration of ethanol into the visceral nerve. The puncture target through the transdiscal approach is located in the transdiscal, and the puncture path is feasible even if the splanchnic nerve is invaded by tumor cells. This puncture path does not pass through the paraspinal tissue, is far from the location of the pancreatic focus, is less affected by tumor cell infiltration and destruction of the structure and is not blocked by the transverse process and ribs; surgical indications increased significantly. For the paraspinal approach, the upper or lower of the vertebral body is a safer path to avoid injury to the intercostal artery, Adamkiewicz artery, and vertebral segmental artery. Therefore, the transdiscal approach reduces the risk of injury to the significant segmental arteries near the vertebral body. Because the paraspinal approach is close to the pleura, in order to avoid the risk of pneumothorax caused by pleura injury, it is necessary to constantly adjust the angle and depth of the puncture needle under DSA exposure and, if necessary, pull out the needle core and inject normal saline to move the lung tissue to 1 side. This process is called water separation. Although the technology of water separation avoids pleural injury, it reduces the concentration of ethanol. In the case of consistent diffusion distribution in theory, the block of high concentration is more complete, and can avoid the recurrence of pain caused by nerve fiber regeneration.^[23]^ In summary, the paraspinal approach should be punctured carefully under DSA multiple exposure, which makes the puncture time and exposure times higher than that of the transdiscal approach.

The VAS scores of the 2 groups after operation were significantly lower than those before, and there was no difference between the 2 groups Ahmed et al.^[12]^ A study through the paraspinal approach damage splanchnic nerve showed that more than 50% relief in pain intensity was obtained in all patients, and more than 50% decrease in opioid dose was observed in >75% of patients at 1 month. Furthermore, there was improvement in both functional status and quality of life in all patients. Puncture effectiveness is 100%. Huacheng et al^[20]^ results of 65 patients with transdiscal approach damage splanchnic nerve showed that 31 patients achieved successful puncture at 1 time, and 3 patients achieved satisfactory results after re-puncture due to unsatisfactory contrast agent distribution. Puncture effectiveness is 95.4%. The NRS score 1 week after the operation was 2.6 ± 0.7, which was lower than the preoperative score (7.6 ± 2.1) (*P* <.001) and was maintained at a low level by the final 2 months follow-up. Similarly, more than 50% relief in pain intensity was obtained in all patients. Our findings are similar to theirs. The 2 approaches can effectively relieve pain in patients. In addition, the 2 approaches had no significant difference in complications. Rosland^[21]^ reports that a transdiscal approach can avoid potential pleural damage and drug reflux to the transdiscal foramen or psoas major muscle and reduce the risk of intraoperative bleeding. Besides, the puncture path is far from the location of the pancreatic lesion. At the same time, the surrounding tissue is relatively less invaded by the tumor, which can reduce the risk of intraoperative bleeding. However, our findings showed no severe complications in the 2 approaches, which may be related to the small sample size in this study and the accurate location of the needle tip by DSA, improving surgical safety.

Although transdiscal puncture is simple, safe, and effective in treating pain from advanced pancreatic cancer, we did not evaluate the risk of discitis and disc herniation caused by transdiscal puncture. Besides, the role of the transdiscal approach in patients with severe thoracolumbar degenerative diseases and calcification of the anterior longitudinal ligament is limited. Therefore, there is a need for long-term studies using a large sample in later stages.

In summary, our research results show that compared with the paraspinal group, the transdiscal group has accurate positioning and simple operation in treating advanced pancreatic cancer pain, can shorten the operation time, and reduce the harm caused by radiation exposure to doctors and patients.

## 5. Conclusions

SNB paraspinal had a significant effect on the treatment of advanced pain in pancreatic cancer patients. However, the transdiscal approach was equally effective, and the positioning is more accurate, and the operation is simpler. Therefore, in patients suitable for this kind of surgery, transdiscal is recommended to reduce radiation and improve surgical tolerance.

## Acknowledgments

The authors thank all Department of Pain Medicine members of the First Affiliated Hospital of Nanchang University.

## Author contributions

**Study design:** Xuexue Zhang, Daying Zhang.

**Data collection:** Xuexue Zhang, Daying Zhang.

**Data analysis:** Yi Yan, Mengye Zhu, Mizhen Qiu. 

**Writing – original draft:** Lingyun Luo, Xintian Cao.
